# Supplementary material for: Metal-to-insulator switching in quantum anomalous Hall states
Source: Nat Commun. 2015 Oct 7;6:8474. doi: 10.1038/ncomms9474 (PMC4633736; doi:10.1038/ncomms9474)
Supplement: Supplementary Information — Supplementary Figures 1-8, Supplementary Notes 1-8 and Supplementary References [file ncomms9474-s1.pdf]

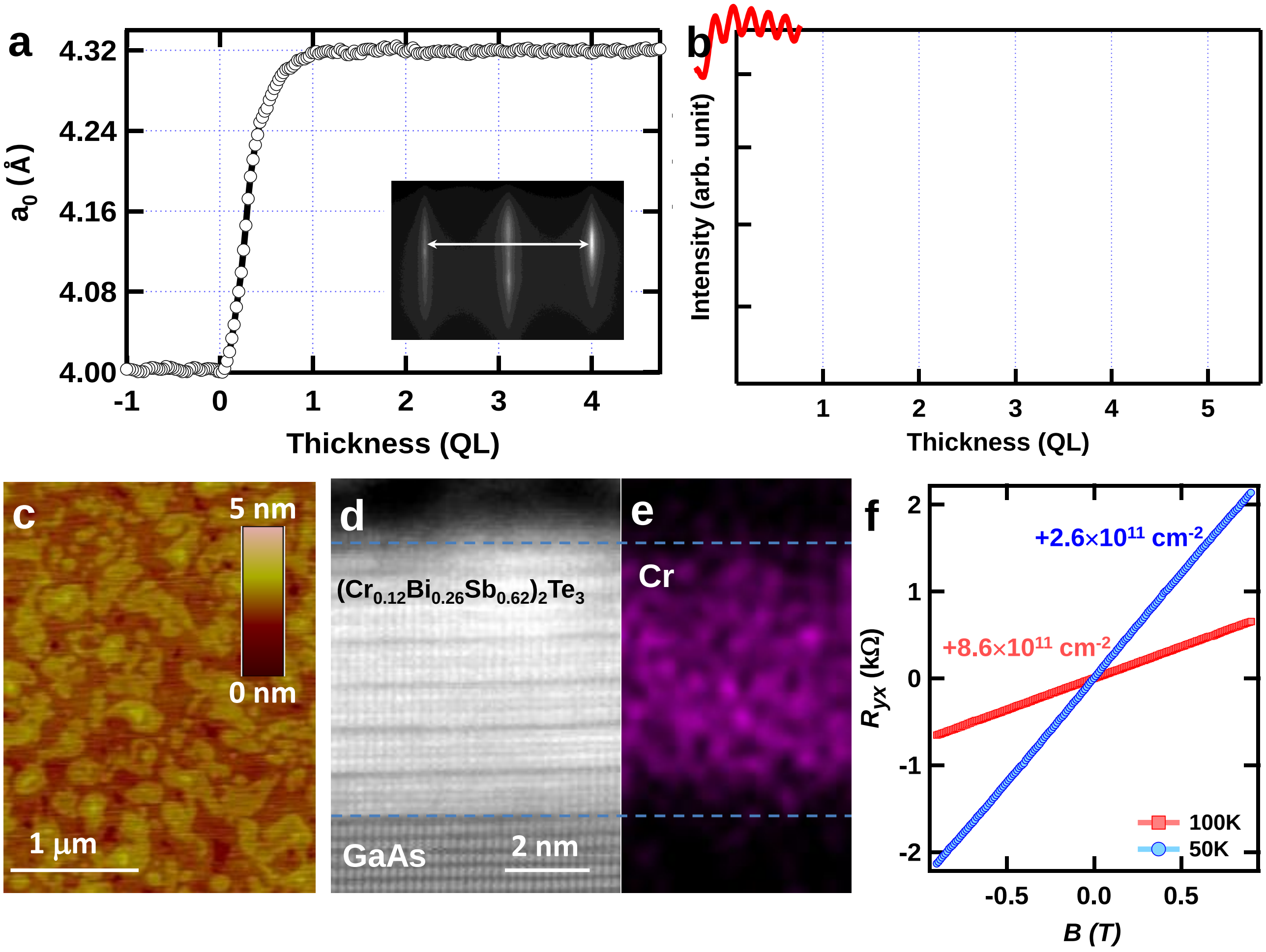

Supplementary Figure 1 | Characterizations of MBE-grown Cr-doped $(Bi_xSb_{1-x})_2Te_3$ film. a. The evolution of lattice constant during the 6 QL $(Cr_{0.12}Bi_{0.26}Sb_{0.62})_2Te_3$ film growth. The lattice relaxation happens within the first QL, which is consistent with the van der Waals growth mode. Inset: RHEED images of the thin film grown on the GaAs (111)B substrate. The *d*-spacing between the two 1st order streaky lines (indicated by the double arrows) is used to estimate the film lattice constant. **b.** RHEED intensity oscillations of the specular peak. **c.** AFM image of the Cr-doped TI thin film with the size of 3 μm × 2 μm. **d.** Cross-sectional high-resolution STEM image, illustrating the crystalline structure of the 6 QL $(Cr_{0.12}Bi_{0.26}Sb_{0.62})_2Te_3$ on the GaAs substrate with sharp interface. **e.** The EDX mapping confirms that the Cr dopants distribute uniformly inside the TI layer. **f.** Hall resistance R_{yx} of the 6QL magnetic TI thin film above Curie temperature. The carrier densities extracted from the linear slopes are $n_{2D} = +2.6 \times 10^{11}$ cm^{-2} at 50 K and $n_{2D} = +8.6 \times 10^{11}$ cm^{-2} at 100 K, respectively.

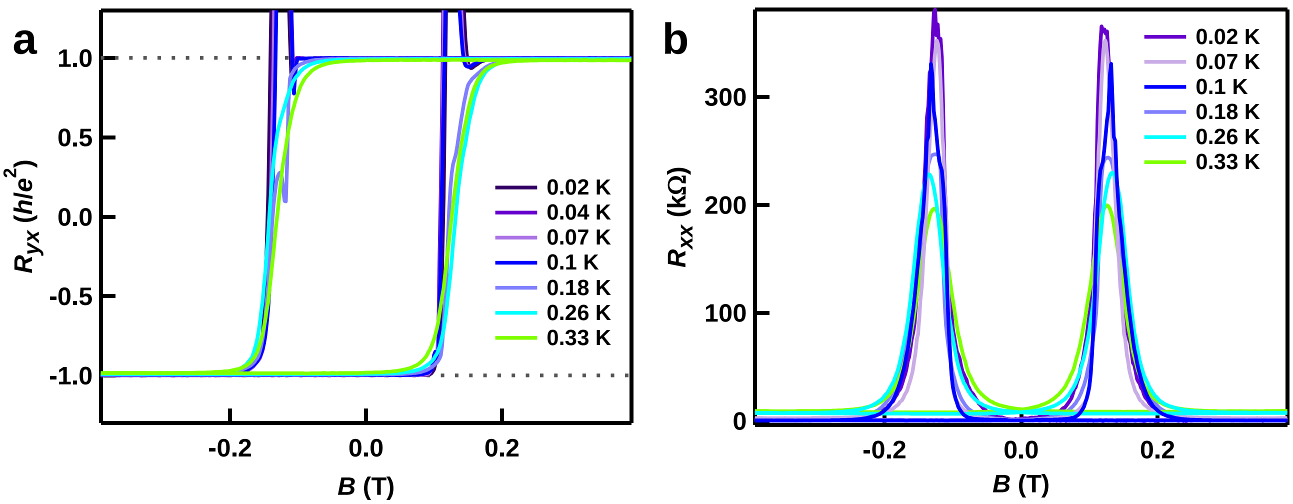

Supplementary Figure 2 | The complete magneto-transport data of a. anomalous Hall resistance R_{yx}, and **b.** longitudinal resistance R_{xx} for the 6 QL $(Cr_{0.12}Bi_{0.26}Sb_{0.62})_2Te_3$ film. The experiments were carried out at the at the SCM1 cell in NHMFL, Tallahassee, USA. The temperature range is [0.02 K, 0.33 K].

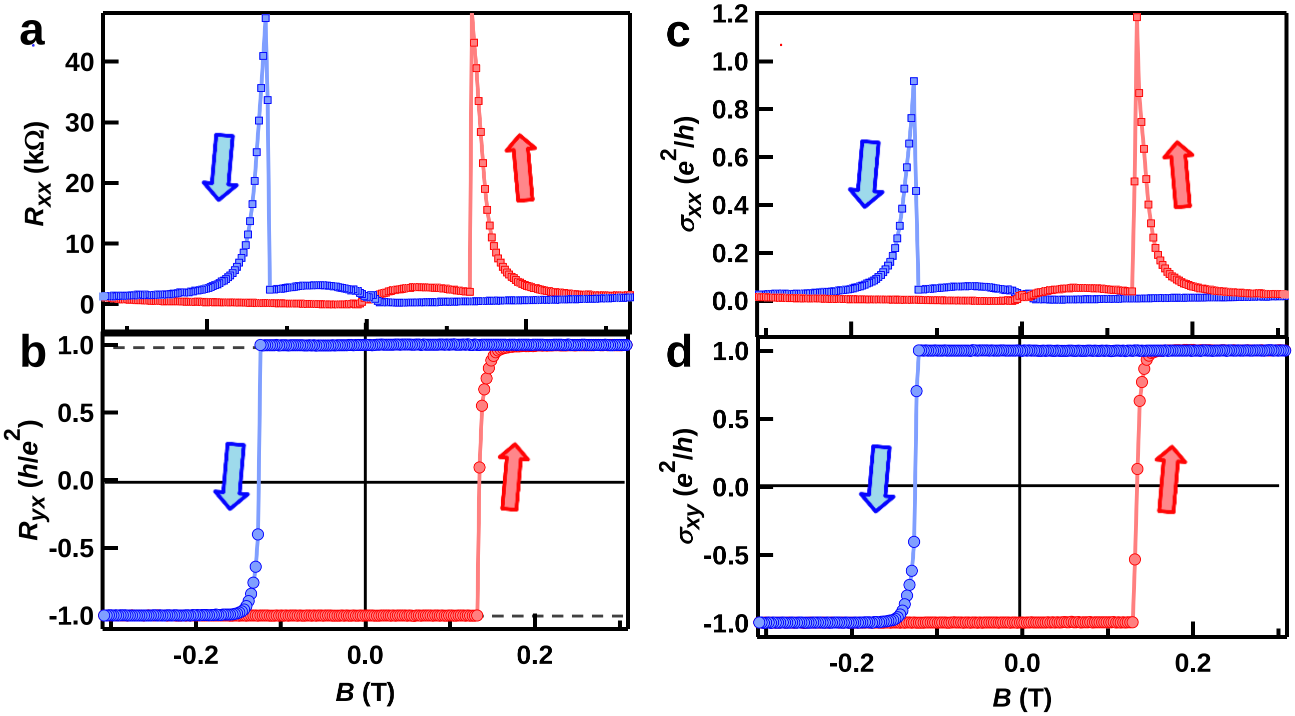

Supplementary Figure 3 | QAHE data of the 10 QL $(Cr_{0.12}Bi_{0.26}Sb_{0.62})_2Te_3$ film used in Ref. [1]. **a-b.** Field-dependent resistance R_{xx} and R_{yx} at 85 mK. **c-b.** Field-dependent conductance σ_{xx} and σ_{xy} at 85 mK. The blue and red arrows indicate the sweeping directions of the applied magnetic field, respectively.

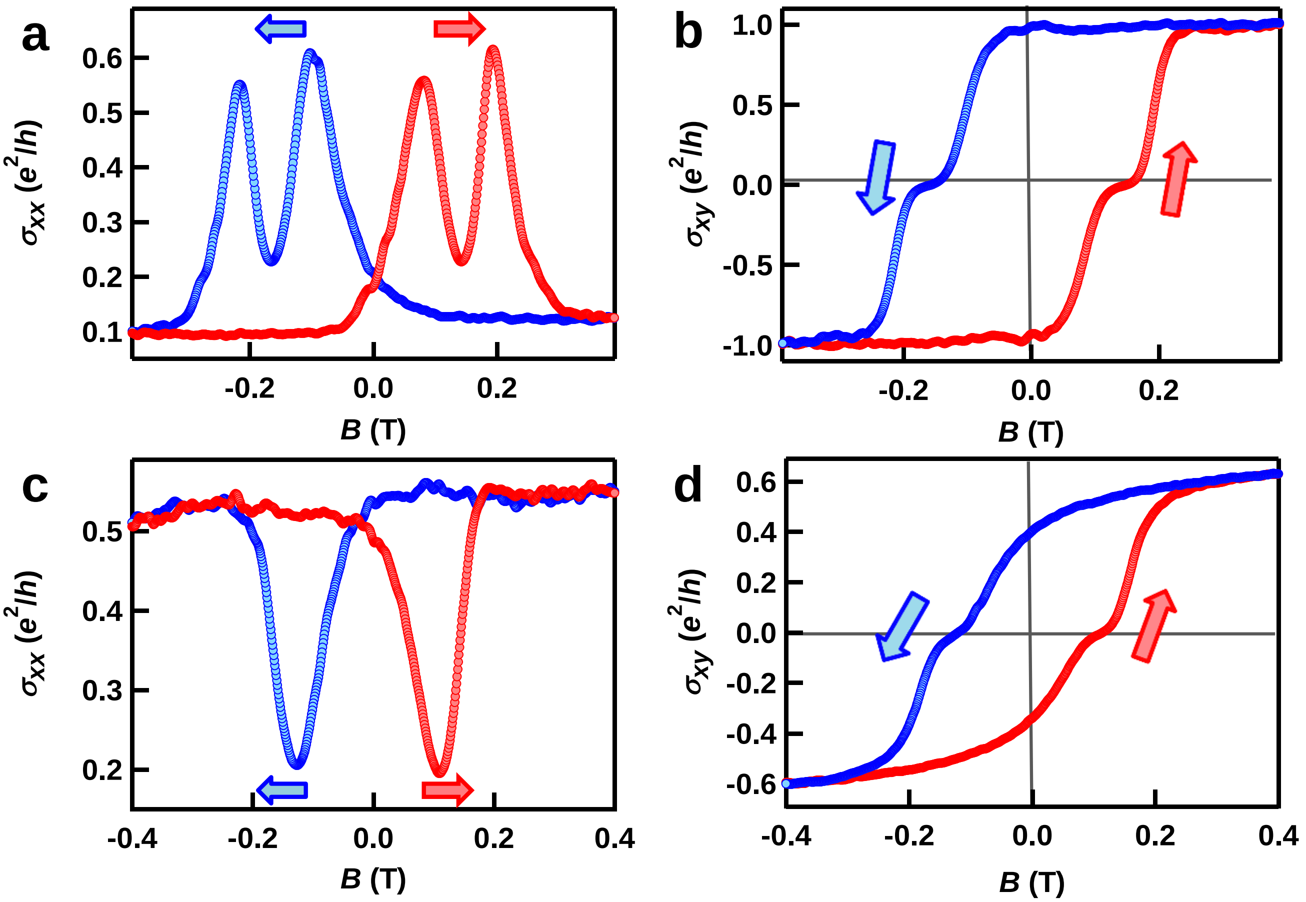

Supplementary Figure 4 | Observation of the zero Hall plateau in a-b. the 6 QL $(Cr_{0.15}Bi_{0.25}Sb_{0.6})_2Te_3$ thin film (T = 77 mK), and **c-d.** the 6 QL $(Cr_{0.1}Bi_{0.27}Sb_{0.63})_2Te_3$ thin film (T = 85 mK). The blue and red arrows indicate the sweeping directions of the applied magnetic field, respectively.

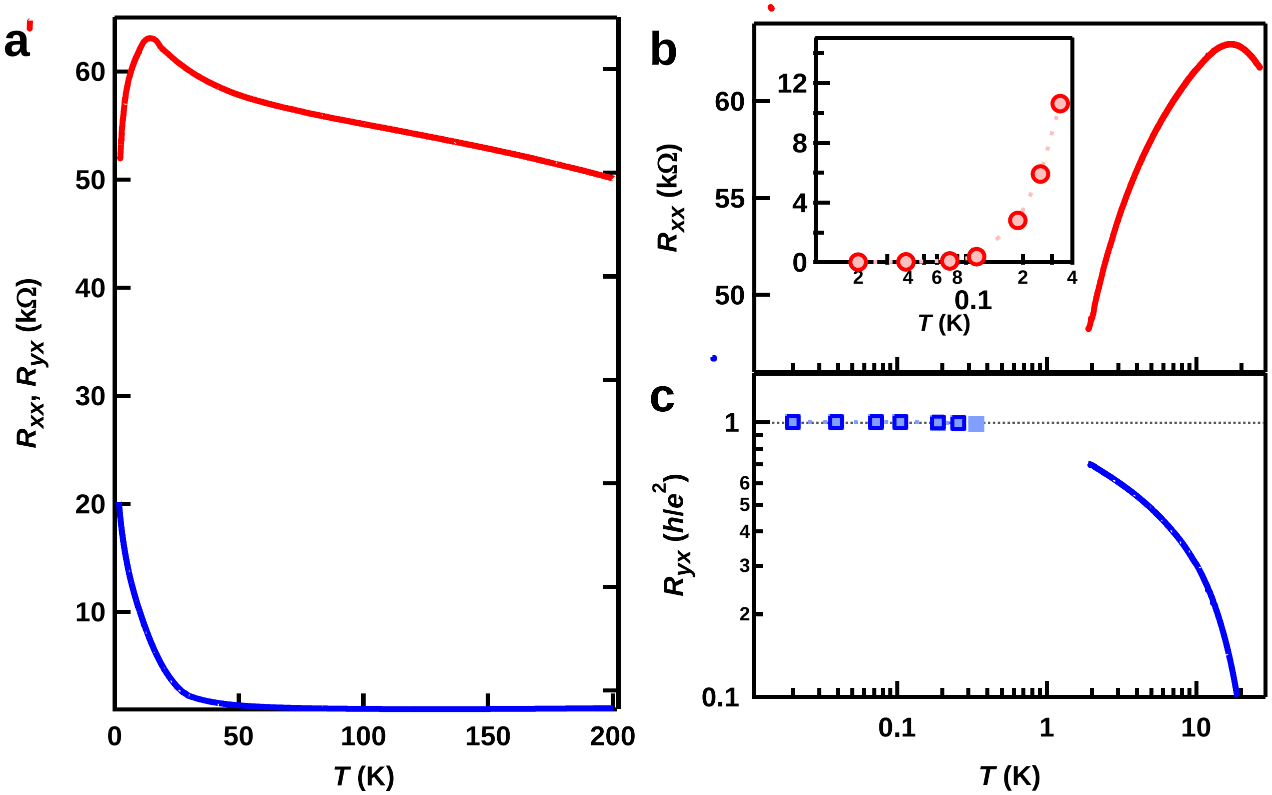

Supplementary Figure 5 | Temperature-dependent transport results. a. Temperature-dependent longitudinal (R_{xx}) and anomalous Hall (R_{yx}) resistances of the 6 QL $(Cr_{0.12}Bi_{0.26}Sb_{0.62})_2Te_3$ film. The applied magnetic field is fixed at B = 1 T. **b.** Temperature-dependent R_{xx} at low temperatures. When T < 15 K, R_{xx}(1T) starts to decrease. **c.**Temperature-dependent R_{yx} in the logarithmic plot. When T < 5 K, the R_{yx} - T curve follows a single power law behavior.

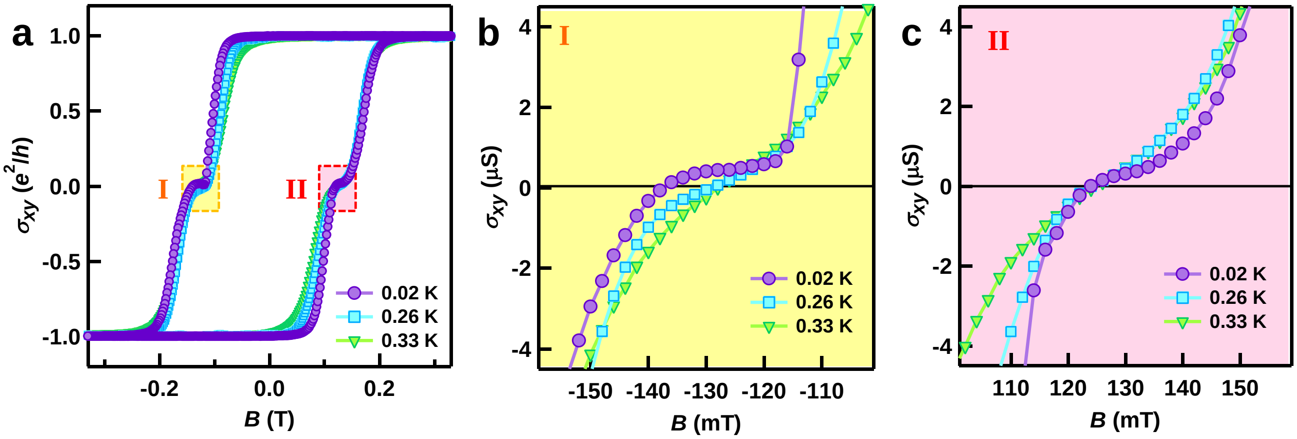

Supplementary Figure 6 | Zoom-in data of the zero-Hall plateau. **a.** Full-scaled temperature-dependent σ_{xy} results as shown in the manuscript. Zoom-in data around the coercive fields **b.** [-0.16 T, -0.1 T] and **c.** [+0.1 T, +0.16 T] to manifest the zero-Hall plateau of the 6 QL $(Cr_{0.12}Bi_{0.26}Sb_{0.62})_2Te_3$ film.

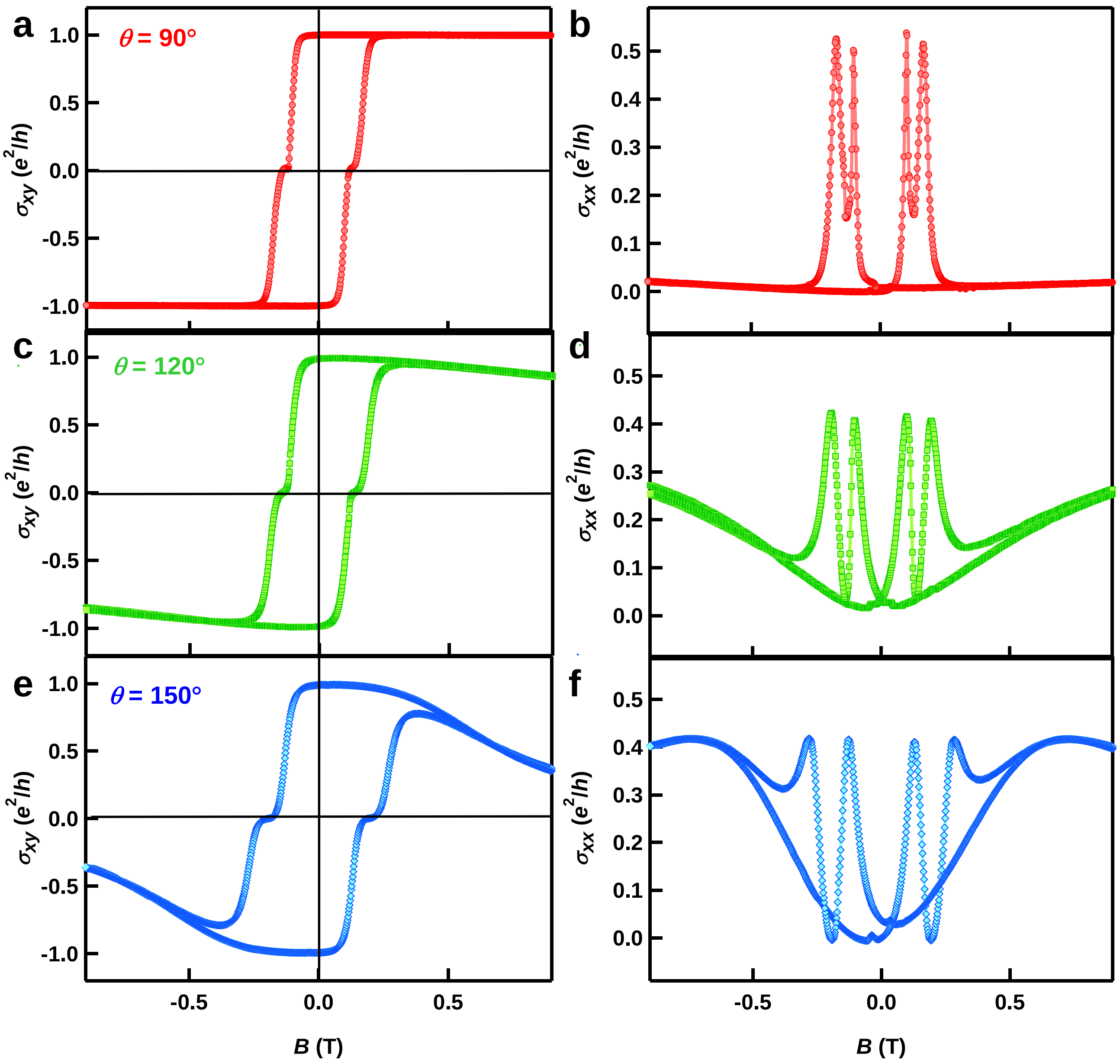

Supplementary Figure 7 | Magneto-conductance σ_{xy} and σ_{xx} of the 6 QL $(Cr_{0.12}Bi_{0.26}Sb_{0.62})_2Te_3$ film at a-b. θ = 90° (same as Figs. 2a - b), **c-d.** θ = 120°, and **e-f.** θ = 150°. Corresponding magneto-resistance results are shown in the manuscript. The hysteresis σ_{xy} curves at all these three tilted angles develop the zero Hall plateau feature, and σ_{xx} show the double-split peaks as well.

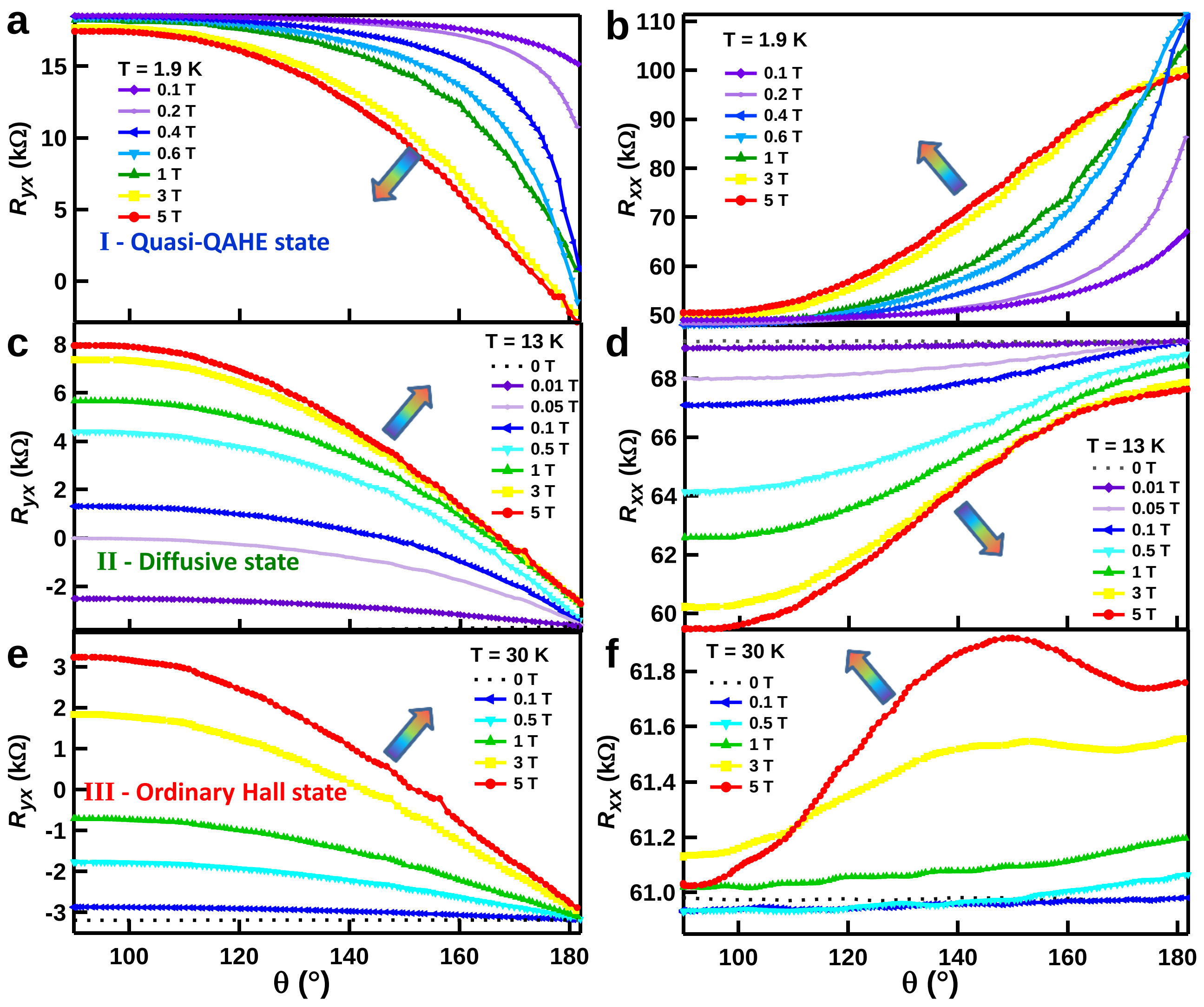

Supplementary Figure 8 | Angle-dependent transport results of R_{yx} and R_{xx} under different fixed B at a-b. T = 1.9 K, **c-d.** T = 13 K, and **e-f.** T = 30 K, respectively.

Supplementary Note 1 | High-quality Cr-doped $(Bi_xSb_{1-x})_2Te_3$ film grown by MBE

In this manuscript, Cr-doped $(Bi_xSb_{1-x})_2Te_3$ films were performed in an ultra-high vacuum Perkin-Elmer MBE system. Semi-insulating ($\rho > 10^6$ Ω·cm) GaAs (111)B substrates were cleaned by acetone with ultrasonic for 10 minutes before loaded into the growth chamber. Then the substrates were annealed to 580°C to remove the native oxide, under Se rich environment, as displayed in the Supplementary Fig. 1a[2]. High-purity Bi (99.9999%), Te (99.9999%), Cr (99.99%) and Sb (99.999%) were evaporated by Knudsen effusion cells. During the growth, the GaAs substrate was maintained around 200 °C (growth temperature), with the Bi, Sb, Te, and Cr shutters opened at the same time. Epitaxial growth was monitored by an in-situ RHEED technique, where the digital RHEED images were captured using a KSA400 system built by K-space Associates, Inc., and the as-grown surface configuration was traced by using the *d*-spacing evolution between the two first-order diffraction lines as shown in the inset of Supplementary Fig. 1a[2]. It can be clearly seen that the lattice relaxation happens within the first QL, indicating that the surface transition from the pristine GaAs to the Cr-doped $(Bi_xSb_{1-x})_2Te_3$ has completed immediately after the formation of the first quintuple layer. After the film growth, a 2 nm Al was evaporated to passivate the surface at room temperature. Al film was later naturally oxidized to form Al_2O_3 after the sample was taken out of the chamber, and such oxide layer effectively prevented the grown magnetic TI film from unwanted environment doping and aging effect[3].

The smooth surface morphology of the 6QL $(Cr_{0.12}Bi_{0.26}Sb_{0.62})_2Te_3$ film used in this manuscript was confirmed by atomic force microscopy (AFM), as shown in Supplementary Fig. 1c where typical TI terraces were well-preserved without any Cr aggregations or clusters, and the surface roughness was as small as ~0.12 nm. In the meantime, high-resolution scanning transmission electron microscopy (HRSTEM) was used to characterize the film structure and crystalline configuration. Supplementary Fig. 1d highlights the highly ordered hexagonal structure of the 6 QL film with an atomically sharp interface on top of the GaAs substrate (i.e., there is no parasitic interfacial layer), and the uniform Cr distribution

inside the host TI matrix is also confirmed by the energy dispersive x-ray (EDX) spectrum, as shown in Supplementary Fig. 1e (the chemical composition of the sample is also determined by the EDX measurement as described in our previous publication[4]). Additionally, temperature-dependent magneto-transport measurements were carried out to study the electrical properties of the 6 QL $(Cr_{0.12}Bi_{0.26}Sb_{0.62})_2Te_3$ sample. In particular, when the temperature is above the Curie temperature ($T_C \sim 30$ K), the ordinary Hall resistance is expressed as $R_{xy} = R_0 \cdot H$, where the slope R_0 is inversely proportional to the Hall density (n_{2D}). Supplementary Figure 1f shows the Hall signals at 50 K (blue) and 100 K (red). The corresponding Hall densities extracted from the linear slopes are $n_{2D} = +2.6 \times 10^{11}$ cm^{-2} at 50 K and $n_{2D} = +8.6 \times 10^{11}$ cm^{-2} at 100 K, respectively, which are much smaller than other reported QAHE samples[1, 5-7], and are comparable with undoped $(Bi_xSb_{1-x})_2Te_3$ samples[2, 8-11].

In summary, by using the MBE growth technique, we are able to prepare high-quality Cr-doped $(Bi_xSb_{1-x})_2Te_3$ thin films in terms of single-crystallinity, well-defined film thickness, smooth surface, sharp interface, uniform Cr doping profile, and most importantly, extremely low bulk carrier density. As shown in the manuscript, such salient features indeed enabled us to observe QAHE and its unique metal-to-insulator transition in the 6 QL $(Cr_{0.12}Bi_{0.26}Sb_{0.62})_2Te_3$ film up to 0.3 K.

Supplementary Note 2 | Complete set of the QAHE data of the 6 QL $(Cr_{0.12}Bi_{0.26}Sb_{0.62})_2Te_3$ film

Supplementary Figure 2 shows the complete magneto-transport results of the 6 QL $(Cr_{0.12}Bi_{0.26}Sb_{0.62})_2Te_3$ film in the QAHE regime (0.02 K – 0.33 K). It can be seen that when $T < 0.1$ K, the hysteresis loops of R_{yx} are almost identical to each other. On the other hand, when $T > 0.1$ K, although the saturated R_{yx} remains at the same quantized value of $\pm\, h/e^2$, the shapes of the hysteresis windows are different. It is known that the strength of the ferromagnetic moment becomes less pronounced at higher temperatures. Under such circumstances, the random multi-domain configuration is more easily to form during the magnetization reversal process between the two $\pm\, h/e^2$ quantized states. Consequently, the transition slope of R_{yx} is expected to be smoother in the $0.1\text{ K} < T < 0.33$ K regime, consistent with the temperature-dependent σ_{xy} slope change as exhibited in Fig. 2d.

Supplementary Note 3 | Quantum anomalous Hall results of the 10 QL $(Cr_{0.12}Bi_{0.26}Sb_{0.62})_2Te_3$ film

In the manuscript, we argue that the phase transition from the $C_1 = 1$ QAHE state to the $C_1 = 0$ insulating state in the 6 QL $(Cr_{0.12}Bi_{0.26}Sb_{0.62})_2Te_3$ film is the result of the competition between the FM exchange field gap (Δ_M) and the quantum confinement-induced hybridization gap (m_0). To further verify the intrinsic relation between the zero Hall plateau and the band topology, Supplementary Fig. 3 shows the QAHE data of the 10 QL $(Cr_{0.12}Bi_{0.26}Sb_{0.62})_2Te_3$ film (i.e., the same chemical compositions and Cr doping level, but different film thickness) which we presented in our previous publication[1]. In contrast to the 6 QL sample (Fig. 2), it is clearly seen that the transition between the two QAHE states ($\sigma_{xy} = \pm e^2/h$) is sharp in the 10 QL film, and there is no intermediate zero Hall plateau during the magnetization reversal process; in the meanwhile, σ_{xx} does not develop any double-peaks around H_C. Given the fact that the top and bottom surfaces are well-separated in the 10 QL sample (i.e., $m_0 = 0$), it is expected that the total Chern number of this system is always non-zero (*i.e.,* no band topology change), and thus the chiral edge conductance is $\sigma_{xy} = C_1 \cdot e^2/h = \pm e^2/h$ (i.e., no zero Hall plateau)[12, 13].

Consequently, our experimental QAHE results on both the 6 QL and 10 QL films $(Cr_{0.12}Bi_{0.26}Sb_{0.62})_2Te_3$ indicate that the QAHE insulating state is indeed a band gap insulating state, in which the gap is attributed to the hybridization of the top and bottom surface states, and the magnetic exchange field is suppressed around H_C (whereas for the quantum Hall effect, the system always crosses a metallic regime from one QHE state to another, including the states of zero Chern number).

Supplementary Note 4 | Observation of zero-Hall plateau in other 6 QL Cr-doped $(Bi_xSb_{1-x})_2Te_3$ samples

In addition to the 6 QL $(Cr_{0.12}Bi_{0.26}Sb_{0.62})_2Te_3$ sample we discussed in the manuscript, we also observe the unique e^2/h-to-zero QAHE plateau transition feature in other 6 QL Cr-doped $(Bi_xSb_{1-x})_2Te_3$ samples. In particular, Supplementary Figs. 4a and b show the field-dependent σ_{xx} and σ_{xy} data in the QAHE state (T = 77 mK) for another 6 QL $(Cr_{0.15}Bi_{0.25}Sb_{0.6})_2Te_3$ thin film which has a slightly higher Cr doping level. It can be seen that both the well-defined Hall plateau with $\sigma_{xy} \sim 0$ and the double-peaked σ_{xx} hysteresis loops are clearly developed around the coercive field ($H_C = \pm0.14$ T) where $|\Delta_M|$ is smaller than $|m_0|$, and the corresponding zero Chern state is achieved. Furthermore, we also observe zero Hall plateau on one "less-ideal" 6 QL $(Cr_{0.1}Bi_{0.27}Sb_{0.63})_2Te_3$ sample. Due to the presence of additional dissipative non-chiral conduction channel (i.e., possible caused by the higher bulk defect density in this sample), the longitudinal resistance R_{xx} (~ 50 kΩ) cannot be fully suppressed at 85 mK. As a result, even though the measured R_{yx} is almost quantized (~ 95% h/e^2), the converted σ_{xy} is not saturated at $\pm\, e^2/h$ when the sample is fully magnetized, as shown in Supplementary Fig. 4b. Nevertheless, the two intermediate Hall plateau across zero around the coercive fields of $H_C = \pm0.11$ T are still observed, similar to other reported data by Y. Feng *et al*[7]. Consequently, we may conclude that as long as the hybridization gap is present, the first Chern number change and its related zero-Hall plateau could always be realized.

Supplementary Note 5 | Temperature-dependence of longitudinal and anomalous Hall resistances

The temperature-dependent (1.9K < T < 200 K) longitudinal (R_{xx}) and anomalous Hall (R_{yx}) resistances of the 6 QL $(Cr_{0.12}Bi_{0.26}Sb_{0.62})_2Te_3$ film were measured in the ppms system. In the diffusive transport region (T > 15 K), the 6 QL $(Cr_{0.12}Bi_{0.26}Sb_{0.62})_2Te_3$ film shows a typical semiconductor behavior, where the sample resistance monotonically increases as the sample temperature drops from 200 K to 1.9 K (solid, red line of Supplementary Fig. 5a) indicating that the Fermi level is inside the bulk band gap. Besides, the Curie temperature (T_C) is found to be around 30 K from the temperature-dependent R_{yx} when the applied magnetic field is fixed at B = 1 T. With both the enhanced magnetization and the reduced thermal activations at lower temperatures, it is seen that the system is driven towards the chiral edge conduction regime when T < 15 K. As a result, R_{xx} starts to decrease rapidly as the sample temperature drops, which is opposite to the $R_{xx} - T$ relation in the higher temperature region (15 K < T < 200 K). On the contrary, the Hall resistance R_{yx} keeps increasing, and finally reaches the quantized h/e^2 when T < 0.33 K, as highlighted in Supplementary Fig. 5c.

Supplementary Note 6 | Zoom-in data of the zero-Hall plateau of the 6 QL sample

In the manuscript, we showed the presence of the zero Hall plateau of the 6 QL $(Cr_{0.12}Bi_{0.26}Sb_{0.62})_2Te_3$ film up to 0.33 K (Fig. 2a). To quantitatively investigate such unique feature, here we show the zoom-in data of Fig. 2a around the coercive fields (i.e., the QAHE phase transition regimes). As displayed in Supplementary Figs. 6b and c, the observed QAHE insulating state in fact exhibits a linear slope with the values of σ_{xy} below 1 μS instead of an ideal "zero plateau". This is due to the fact that the measured longitudinal resistance R_{xx} is always finite and the Hall resistance R_{yx} only becomes zero at a specific magnetic field. However, we should point out that the e^2/h-to-zero QAHE plateau transition is still manifested by the dramatic change of the σ_{xy} slope $S = \partial\sigma_{xy} / \partial H$. For example, the slope of the QAHE insulating state is found to be 2.02×10^{-9} S/Oe at T = 0.02 K, and is 2 order of magnitude smaller than the transition slope (S = 2.68×10^{-7} S/Oe) defined in the manuscript (i.e., T = 0.02 K data point of Fig. 2d).

Supplementary Note 7 | Angle-dependent longitudinal and Hall magneto-conductance results

Following the same reciprocal resistance-to-conductance (ρ - σ) tensor conversion that was discussed in the manuscript, we also investigate the angle-dependent transport results (Fig. 3) in the conductance plot. Supplementary Figure 7 exhibits the corresponding data at three different tilted angles θ = 90°, 120°, and 150°, respectively. It can be seen that both the zero-Hall plateau and double-split σ_{xx} still remain obvious under all rotation conditions.

Supplementary Note 8 | Angle-dependent anomalous Hall results at different temperatures

In the manuscript, we discussed the angle-dependent transport results of the 6 QL $(Cr_{0.12}Bi_{0.26}Sb_{0.62})_2Te_3$ film in the QAHE regime (0.02 K – 0.33 K), and obtained the single semicircle QAHE phase diagram in the conductance plot. In order to further study the scaling behavior of such phase diagram, it is equally important to compare Fig. 3 with the angle-dependent data at the non-QAHE regimes. From Supplementary Fig. 5, we can divide the temperature-dependent R_{xx} curve into three regimes: (I) $T < 13$ K, the film is driven towards the quantum transport state; (II) 13 K $< T <$ 30 K, the film is in the diffusive transport state with the presence of anomalous Hall effect; and (III) $T > 30$ K, the film is in the ordinary diffusive transport state without any FM ordering. By performing the same measurements as described in Figs. 3c – d (i.e., record both R_{xx} and R_{yx} when the sample is continuously rotated from 90° to 180° under different fixed applied magnetic fields), we summarize the results in Supplementary Fig. 8 at T = 1.9 K, 13 K, and 30 K, respectively. It can be clearly seen that when the sample is in the quasi-QAHE state (T = 1.9 K), the angle-dependent R_{xx} and R_{yx} curves are quite similar to Figs. 3c - d. Specifically speaking, all R_{yx} curves tend to converge at two fixed points. In contrast, when the diffusive conduction becomes dominant at higher temperature (T = 13 K and 30 K), the dominant ordinary Hall component R_0H strongly depends on the external magnetic field, and the converging feature at $\theta = 90°$ no longer holds. If we further convert Supplementary Fig. 8 into the conductance (σ_{xx}, σ_{xy}) plot, and compare the transition curves at B = 1 T between these three regimes and the QAHE state, the corresponding results are shown in Fig. 4d.

Supplementary References:

1. Kou, X.F. et al. Scale-Invariant Quantum Anomalous Hall Effect in Magnetic Topological Insulators beyond the Two-Dimensional Limit. *Phys Rev Lett* **113**, 137201 (2014).
2. He, L. et al. Evidence of the two surface states of $(Bi_{0.53}Sb_{0.47})_2Te_3$ films grown by van der Waals epitaxy. *Sci. Rep.* **3**, 3406 (2013).
3. Lang, M.R. et al. Revelation of Topological Surface States in Bi2Se3 Thin Films by In Situ Al Passivation. *Acs Nano* **6**, 295-302 (2012).
4. Kou, X.F. et al. Interplay between Different Magnetisms in Cr-Doped Topological Insulators. *Acs Nano* **7**, 9205-9212 (2013).
5. Chang, C.Z. et al. Experimental Observation of the Quantum Anomalous Hall Effect in a Magnetic Topological Insulator. *Science* **340**, 167-170 (2013).
6. Checkelsky, J.G. et al. Trajectory of the anomalous Hall effect towards the quantized state in a ferromagnetic topological insulator. *Nat Phys* **10**, 731-736 (2014).
7. Feng, Y. et al. Observation of the zero Hall plateau in a quantum anomalous Hall insulator. Preprint at http://arxiv.org/abs/1503.04569 (2015).
8. Xu, Y. et al. Observation of topological surface state quantum Hall effect in an intrinsic three-dimensional topological insulator. *Nat Phys* **10**, 956-963 (2014).
9. Kong, D.S. et al. Ambipolar field effect in the ternary topological insulator $(Bi_xSb_{1-x})_2Te_3$ by composition tuning. *Nat Nanotechnol* **6**, 705-709 (2011).
10. Hong, S.S., Cha, J.J., Kong, D.S. & Cui, Y. Ultra-low carrier concentration and surface-dominant transport in antimony-doped Bi_2Se_3 topological insulator nanoribbons. *Nat Commun* **3**, 757 (2012).
11. Zhang, J.S. et al. Band structure engineering in $(Bi_{1-x}Sb_x)_2Te_3$ ternary topological insulators. *Nat Commun* **2:574** (2011).
12. Wang, J., Lian, B. & Zhang, S.C. Universal scaling of the quantum anomalous Hall plateau transition. *Phys Rev B* **89**, 085106 (2014).
13. Lu, H.Z., Zhao, A. & Shen, S.Q. Quantum Transport in Magnetic Topological Insulator Thin Films. *Phys Rev Lett* **111**, 146802 (2013).
